# Bibliometric and visual analysis of fecal microbiota transplantation research from 2012 to 2021

**DOI:** 10.3389/fcimb.2022.1057492

**Published:** 2022-11-10

**Authors:** Fengwei Zhang, Peilin Yang, Yilin Chen, Ruirui Wang, Baocheng Liu, Jianying Wang, Min Yuan, Lei Zhang

**Affiliations:** Shanghai Innovation Center of TCM Health Service, Shanghai University of Traditional Chinese Medicine, Shanghai, China

**Keywords:** fecal microbiota transplantation, gut microbiota, bibliometric, CiteSpace, clostridium difficile infection

## Abstract

**Background:**

Fecal microbiota transplantation (FMT) is an emerging therapy for diseases associated with intestinal flora imbalance that has attracted increasing attention in recent years. This study aims to provide an overview of research trends in the field, and act as a reference point for future scientific research by analyzing the state of current research, identifying hotspots, and potential frontiers of FMT.

**Methods:**

Articles relating to FMT that were published between the years 2012 and 2021 were retrieved from the Web of Science Core Collection. Bibliometric analysis was performed using Microsoft Excel and CiteSpace.

**Results:**

A total of 2,403 English language articles relating to FMT research were published over the last ten years. Most of this research was carried out in the United States of America, with Harvard Medical school being the most productive institution. Much of the research was published in the *PLoS One journal*. Alexander Khoruts was identified as a prominent, productive researcher in the field. Keyword analysis revealed that research hot spots included gut microbiota, *Clostridium difficile* infection (CDI), and diseases. Burst detection indicated that future research frontiers include clinical practice guidelines and strategies.

**Conclusion:**

Our analysis explored hot spots and emerging trends in the FMT field. Indications for use of FMT extended from digestive system diseases to other systemic diseases. Additionally, areas such as risk assessment and control, along with application methods were also a focus of current research. Moreover, research relating to optimization of clinical practice has excellent prospects.

## Introduction

Fecal microbiota transplantation (FMT) is a method for treating intestinal and extra-intestinal diseases. FMT can be used to reconstitute the intestinal microbial community in recipients by transplanting the functional flora in the feces of healthy donors into the gastrointestinal tract of patients (Gupta and Khanna, 2017; Wang et al., 2019b). Historical records revealed that feces treatment occurred in China as early as the fourth century AD. In 1958, Eiseman published his first paper on the use of FMT therapy to treat pseudomembranous colitis (Eiseman et al., 1958). Studies have shown that FMT has a definite therapeutic effect on several gastrointestinal diseases including recurrent *Clostridium difficile* (*C. difficile*) infection (CDI) (Cammarota et al., 2017), ulcerative colitis, and Crohn’s disease (Holleran et al., 2018). Moreover, FMT has also been shown to be beneficial in multiple system diseases including cancer (Chen et al., 2019), obesity (Aron-Wisnewsky et al., 2019), and hepatic encephalopathy (Karakan, 2017). In recent years, the field of FMT has enjoyed rapid development with the progress of the human microbiome project (Turnbaugh et al., 2007), the establishment of stool banks (Kragsnaes et al., 2020), and the improvement of transplantation techniques and specifications (Khoruts et al., 2019; Ossorio and Zhou, 2019).

Gut microbiota dysbiosis is involved in the pathology of many diseases (Guarner and Malagelada, 2003; Schmidt et al., 2018). The intestinal flora participates in metabolism and plays a vital role in the host’s immune, nutritional and digestive functions (Ananthakrishnan et al., 2019; Gomaa, 2020; Zhou et al., 2020). Short-term interventions often fail to alter the composition of the flora (Dostal Webster et al., 2019) as the intestinal community is quite resistant to change. In contrast, FMT has significant advantages in establishing healthy microbial systems. FMT reconstructs the intestinal microbial community by means of transplantation and colonization, increasing the number of beneficial bacteria and rebuilding the patient’s homeostatic environment (Suez et al., 2018). FMT was written into the guidelines for the diagnosis, treatment, and prevention of CDI in 2013 (Surawicz et al., 2013). In 2019, the international conference on FMT reached consensuses on stool banking and the implementation rules for fecal transplantation (Cammarota et al., 2019).

As the field of FMT has developed rapidly and shows great potential in treating various diseases, we carried out a bibliometric analysis of relevant articles published between 2012 and 2021. The bibliometric method allows quantitative and qualitative analyses of specific topics and articles to evaluate academic productivity and trends. CiteSpace is a network analysis and visualization software developed by Chaomei Chen based on the JAVA platform (Chen and Chen, 2005). It can identify relationships between scientific articles and explore emerging trends and theme changes over time. This study explored annual trends of publications, countries/regions of high productivity, institutions and authors, core journals, co-cited references, current status and future directions of research.

## Materials and methods

### Data source and search strategy

The literature retrieval was conducted on March 24th, 2022, using the Web of Science Core Collection. The search formula used was “((((((((TS=(fecal microbiota transplantation)) OR TS=(faecal microbiota transplantation)) OR TS=(fecal microbiota transfer)) OR TS=(faecal microbiota transfer)) OR TS=(fecal microbiota transplant)) OR TS=(faecal microbiota transplant)) AND PY=(2012-2021)) AND DT=(Article)) AND LA=(English).” We placed restrictions on the publication date (2012-01-01 to 2021-12-31) and the language (English). Inclusion was limited to original research articles. Other literature types such as review articles, meeting summaries, and editorial materials were excluded. Ultimately, we identified 2,403 eligible records (**Supplementary Figure 1**). Search records were downloaded and exported to CiteSpace software for subsequent analysis.

The journal impact factor (IF) and quartile were obtained from Journal Citation Reports 2021. The IF is determined by the number of citations and total articles in the last two years (Garfield, 2006) and reflects the journal’s influence.

### Data analysis

Microsoft Excel 2016 was used for basic calculations and plotting of annual trends of publications and citations. Publications and citations were represented by bar and line charts. The trend test was performed using polynomial curve fitting.

CiteSpace (5.8. R 3 64-bit) was used for bibliometric and visual analysis. The visual map consists of nodes and links. In the network, the nodes were used to represent the countries, institutions, authors, references, etc. Meanwhile, the size and frequency of the node were in direct proportion. The different colors of nodes correspond to different periods. The connection link between nodes indicates the relationship between them, and the thickness of the connection line indicates the strength of the association. The number of nodes and connections from the network are represented by N and E, respectively. Betweenness centrality was used to measure the centrality of the vertices of the shortest path in a network. It indicated the importance of a node and the relationship between neighboring nodes. Nodes with a betweenness centrality greater than 0.1 were usually marked with purple circles and were considered turning points or key points in the field.

## Results

### Temporal trends of publications and citations

This study included 2,403 English articles related to FMT. A total of 42,796 articles were cited, with a total citation number of 92,290, and an average citation number of 38.41 times. The H-index was 134.

**Figure 1:**
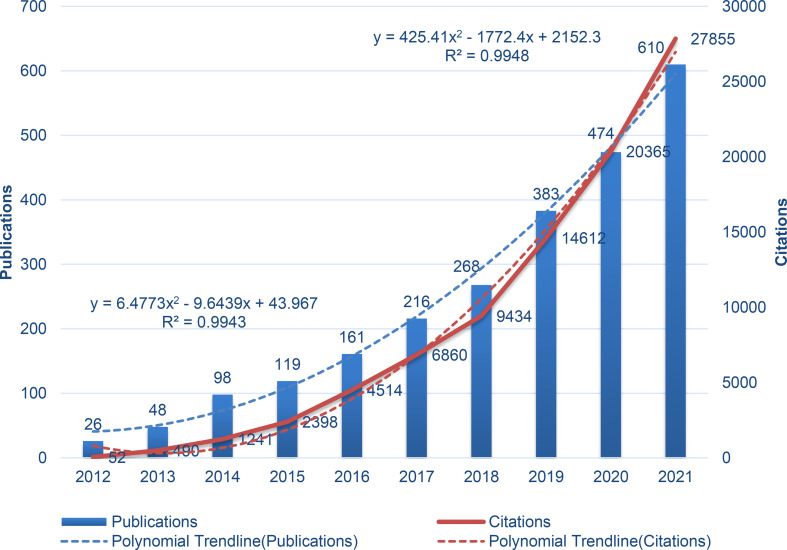
Annual trends in FMT research publications and citations.

The number of FMT publications trended upwards over the period from 2012 to 2021, especially after 2018 (**Figure 1**). In total, 1,467 articles were published from 2019 to 2021, accounting for 61.05% of the total publications over the 10 years. Over the past decade, citations have increased dramatically, especially from 2018 to 2021. The polynomial fitting curve showed significant correlations between publications, citations, and year (R^2^ = 0.9943, R^2^ = 0.9948). The above results demonstrate that FMT research has received increasing attention in recent years. It is expected that the number of related articles and citations will enter a sustained growth stage in the future.

### Countries and institutions analysis

**Figure 2:**
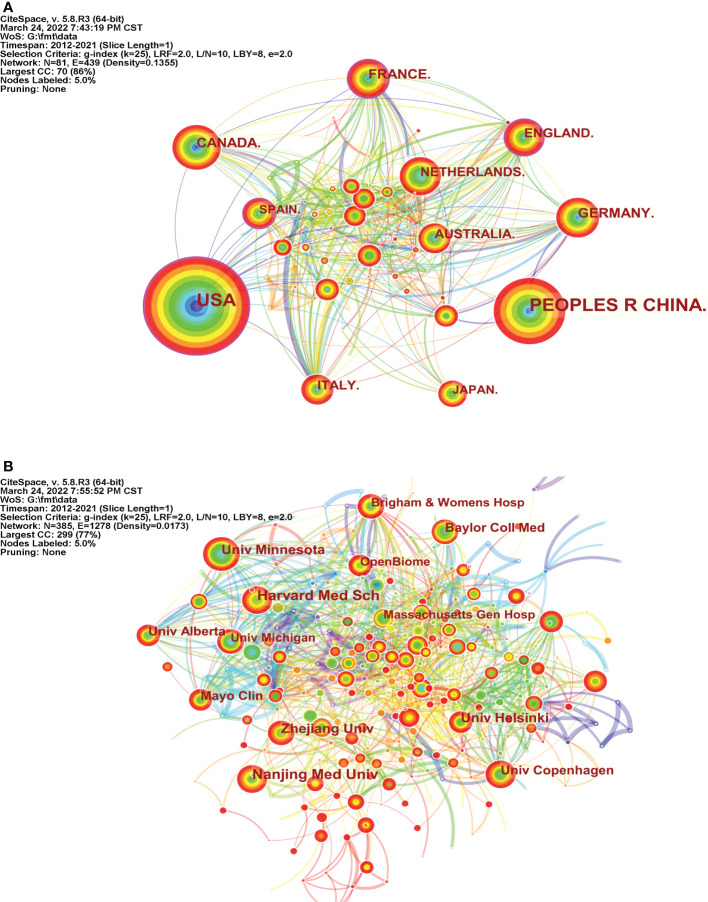
Countries and Institutions Analysis. **(A)** Map of countries performing FMT-related research (N=81, E=439); **(B)** Map of institutes performing FMT-related research (N=385, E=1278).

Scholars in 81 countries published research articles (**Figure 2A**). The top 10 countries are listed in **Table 1**. The United States of America (USA) was the leading country for publications, followed by the People’s Republic of China, Canada, France, and Germany. The top five countries in terms of centrality were France (0.20), England (0.17), the USA (0.16), Spain (0.15), and Germany (0.10), indicating that these countries are more influential and cooperative in FMT research.

**Table 1:** The most productive countries and institutions in FMT research.

Rank	Count	Centrality	Country	Rank	Count	Centrality	Institution
1	914	0.16	USA	1	59	0.01	Harvard Medical School
2	682	0	China	2	57	0	Nanjing Medical University
3	146	0.05	Canada	3	52	0.03	Zhejiang University
4	146	0.2	France	4	49	0.09	University of Minnesota
5	130	0.1	Germany	5	43	0.02	University of Helsinki
6	117	0.17	England	6	40	0.07	University of Copenhagen
7	110	0.05	Italy	7	39	0.03	Baylor College of Medicine
8	103	0.04	Netherlands	8	35	0.04	Mayo Clinic
9	97	0.08	Australia	9	35	0.03	University of Alberta
10	87	0.02	Japan	10	33	0.03	University of Michigan

A total of 385 institutions were enrolled in the FMT study (**Figure 2B**). Five of the top 10 most productive institutions are located in the USA (**Table 1**): Harvard University, University of Minnesota, Baylor College of Medicine, Mayo Clinic, and University of Michigan, indicating that American scientific research institutions are heavily involved in the FMT research field. Notably, OpenBiome contributed 33 articles as a non-profit organization (stool bank) dedicated to collecting, screening, and transporting fecal samples (Cammarota et al., 2019).

The extensive network of cooperation between these countries and institutions indicates the importance of international collaboration in FMT research. Although China has an advantage in the number of publications, disparities still persist in global communication and collaboration compared with developed countries.

### Journal and author analysis

In total, 728 journals were identified as publishing FMT-related articles, and **Table 2** shows the top 10 academic journals. More than 50 articles were published in each of *PLoS One*, *Scientific Reports*, *Frontiers in Microbiology*, and *Gut Microbes*. The mean IF of top 10 journals were 14.192 and the highest IF was 33.883 (*Gastroenterology*). Among these top 10 journals, the fields of gastroenterology & hepatology, and microbiology were represented by five and two journals, respectively, further illustrating that the digestive system is an important area of focus for FMT research. In addition, all the listed journals were distributed in Q1 or Q2, indicating that the above journals had strong academic influences on FMT research.

**Table 2:** Journals with the most published FMT-related articles.

Rank	Count	Journal	IF^1^	Quartile in Category
1	65	PLoS One	3.752	Q2
2	65	Scientific Reports	4.996	Q2
3	54	Frontiers in Microbiology	6.064	Q1
4	54	Gut Microbes	9.434	Q1
5	53	Microbiome	16.837	Q1
6	42	Gut	31.793	Q1
7	38	Gastroenterology	33.883	Q1
8	36	World Journal of Gastroenterology	5.374	Q2
9	33	Clinical Infectious Diseases	20.999	Q1
10	27	Frontiers in Immunology	8.786	Q1

^1^Data from the 2021 edition of Journal Citation Reports.

**Figure 3:**
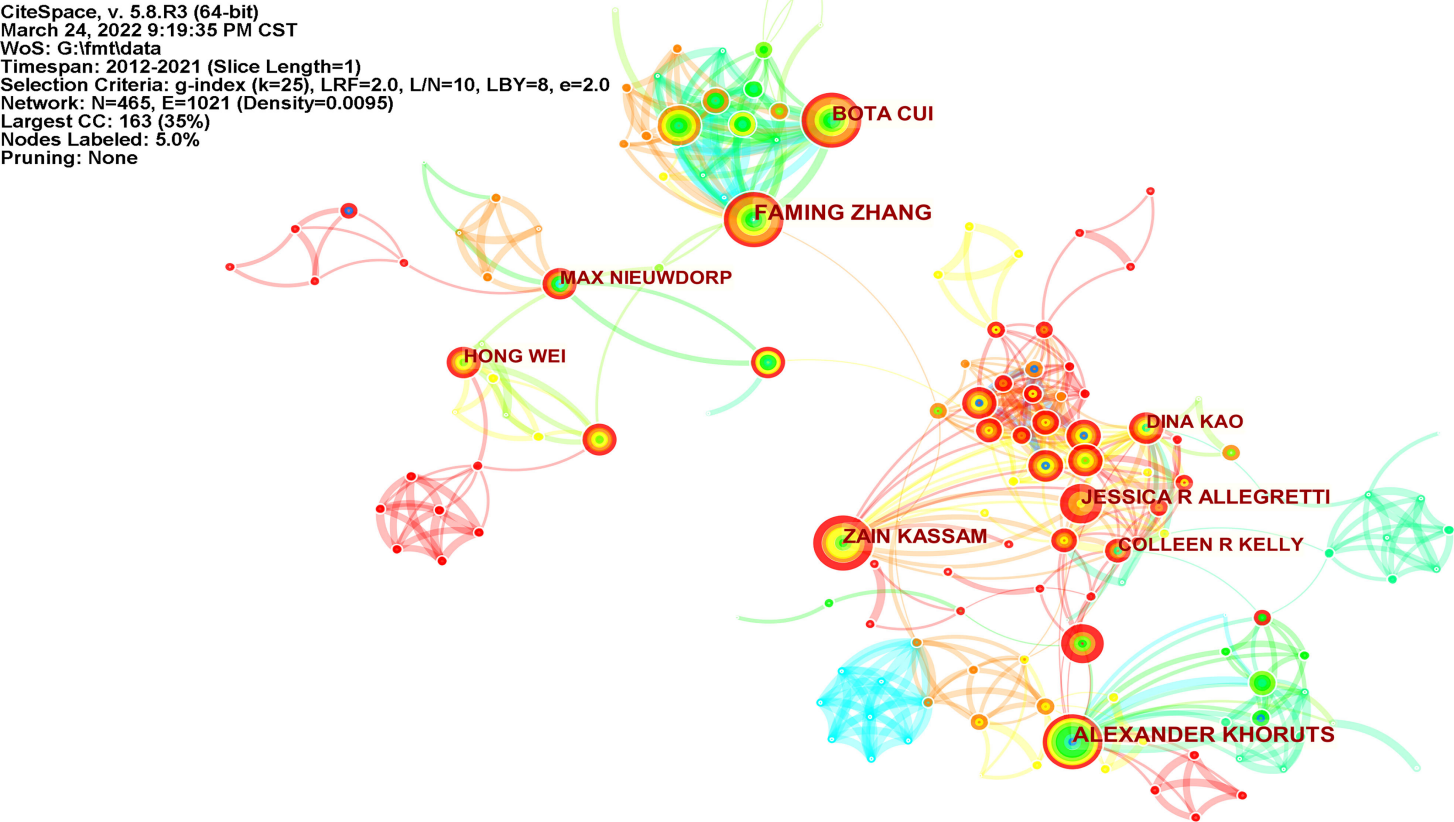
Map of authors performing FMT-related research (N=465, E=1021).

There were 465 authors who published articles related to FMT (**Figure 3**). Among the top 10 most productive authors in **Table 3**, Alexander Khoruts, a gastroenterologist at the University of Minnesota, has published 39 articles. Faming Zhang, who is known as the “first man of FMT in China” and is based at Nanjing Medical University, had a centrality of 0.11. Faming Zhang and Bota Cui cooperated closely together to improve the practices of FMT (Zhang et al., 2020).

**Table 3:** Top 10 productive authors of FMT-related research articles.

Rank	Count	Centrality	Year	Authors
1	39	0.05	2012	Alexander Khoruts
2	35	0.11	2015	Faming Zhang
3	29	0	2015	Bota Cui
4	29	0.01	2017	Zain Kassam
5	27	0	2014	Jessica R Allegretti
6	24	0.05	2014	Dina Kao
7	23	0.02	2018	Hong Wei
8	22	0.02	2014	Colleen R Kelly
9	22	0.03	2015	Max Nieuwdorp
10	21	0	2016	Ting Zhang

### Category analysis

**Figure 4:**
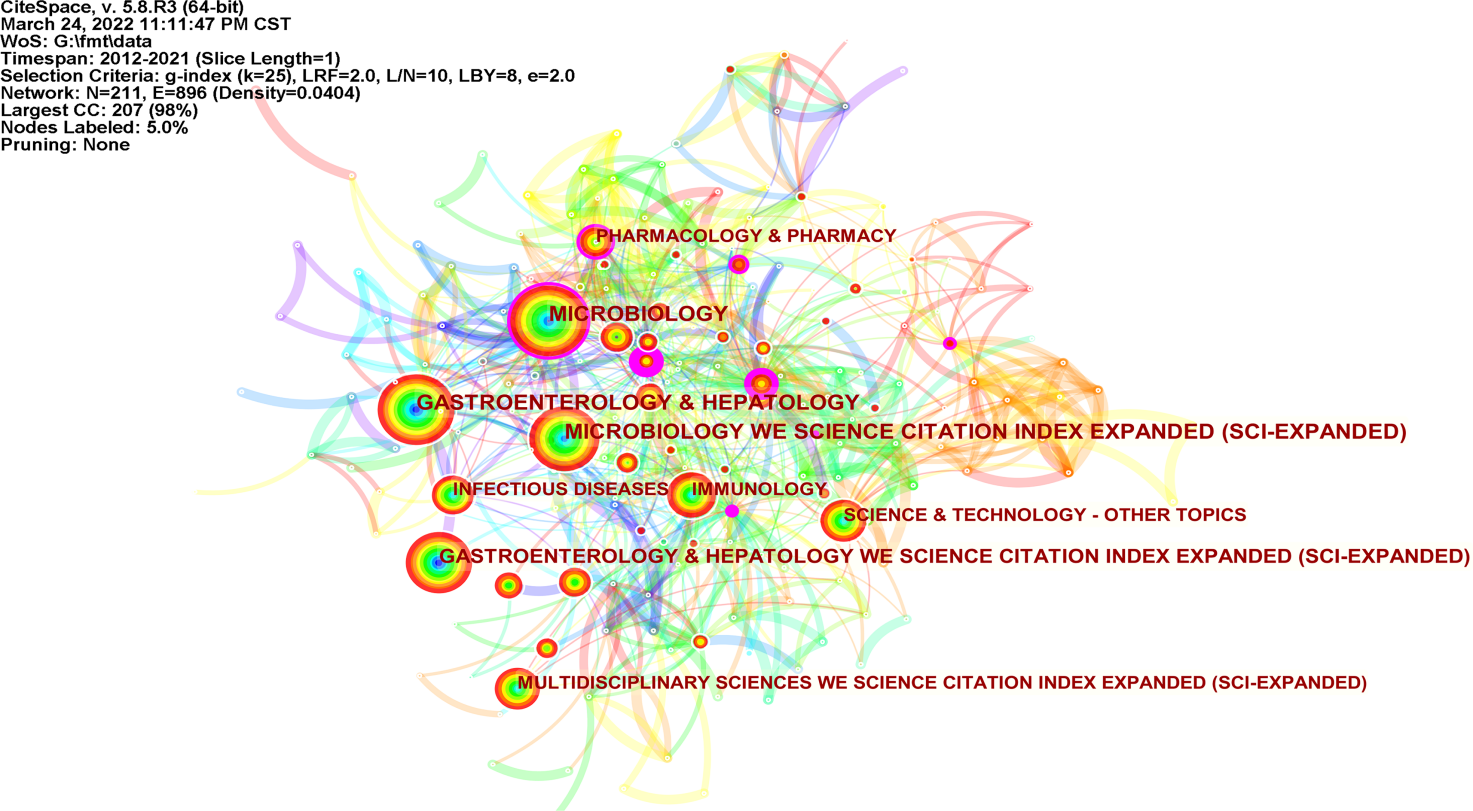
Map of categories related to FMT (N=211, E=896).

According to the category analysis of Web of Science, FMT research mainly focuses on microbiology, gastroenterology, hepatology, and immunology (**Figure 4**). Social Science Citation Index (SSCI) (0.43), Biochemistry & Molecular Biology (0.3), and Conference Procedures Citation Index-Science (CPCI-S) (0.23) had relatively high concentricity.

### Keyword analysis

**Figure 5:**
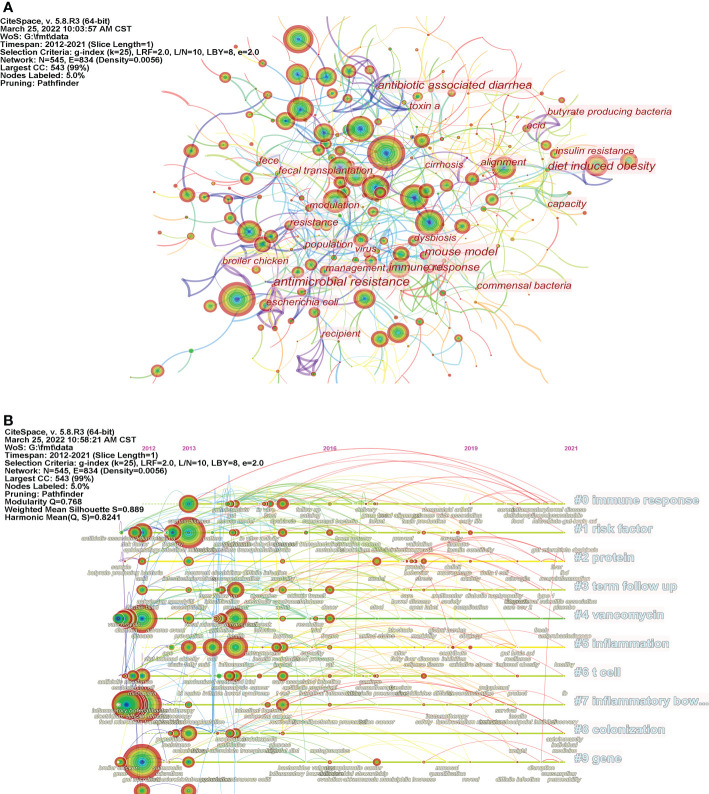
Analysis of keywords. **(A)** Map of keywords related to FMT (N=545, E=834); **(B)** The timeline visualization map of keywords related to FMT.

The keyword co-occurrence visualization map consists of 545 nodes and 1,954 links (**Figure 5A**). The top 5 keywords according to their frequency of occurrence were “gut microbiota,” “fecal microbiota transplantation,” *“clostridium difficile* infection,” “disease” and “infectious bowel disease”, indicating that the damage to the gastrointestinal tract caused by CDI and other inflammatory diseases were hot topics in FMT research. According to the centrality, the top five keywords are “antimicrobial resistance” (0.19), “mouse model” (0.18), “gut” (0.15), “immune response” (0.15), and “diet” (0.14). This indicates that FMT has a potential role in counteracting the side effects of certain drugs and lifestyles, and relevant animal experiments have been widely conducted (**Table 4**). In addition, we conducted clustering analysis on co-occurring keywords. The top 10 clusters with the most significant number of keywords are shown in **Table 4**. The immune response (cluster #0) is the largest cluster, and is considered as one of the main therapeutic of FMT (Frisbee and Petri, 2020). The alteration of host immune responses by FMT has been demonstrated in multiple studies (Jang et al., 2021; Spencer et al., 2021). Furthermore, disruption of the intestinal microbiota by antibiotic therapy (Gregory et al., 2021; Tomkovich et al., 2021) was the primary risk factor for CDI (cluster #1 risk factor). Meanwhile, other risk factors for FMT, such as early clinical recurrence of inflammatory bowel disease, have also received attention (Zhao et al., 2021).

**Table 4:** Top 10 keywords and clusters related to FMT research.

Rank	Count	Centrality	Year	Keywords	ClusterID	Size	Label (LLR)
1	667	0.01	2012	gut microbiota	#0	48	immune response
2	540	0.02	2012	fecal microbiota transplantation	#1	43	risk factor
3	320	0.01	2013	intestinal microbiota	#2	39	protein
4	306	0.04	2012	clostridium difficile infection	#3	37	term follow up
5	271	0.01	2012	disease	#4	34	vancomycin
6	265	0.02	2012	inflammatory bowel disease	#5	32	inflammation
7	231	0.03	2012	ulcerative coliti	#6	31	t cell
8	200	0.01	2014	inflammation	#7	29	inflammatory bowel disease
9	189	0.04	2012	diversity	#8	27	colonization
10	189	0.02	2012	clostridium difficile	#9	27	gut microbiota

The timeline view was used to demonstrate the changing trends of different clusters over time (**Figure 5B**). The first 10 clusters are shown in **Table 4**. “Immune response,” “risk factor,” and “protein” are the clusters that contain the most keywords, while clusters such as “immune response,” “infrared,” and “colonization” appear relatively late. Additionally, people are increasingly concerned about the safety and efficacy of FMT due to the increased development of fecal transplantation technology and the deepening of relevant research.

Burst keyword detection can reveal the dynamic changes in hot spots and research frontiers over time. **Table 5** shows the 30 keywords with the strongest citation bursts in the past decade. From 2012 to 2015, the main focus was on the treatment of diarrhea and *C. difficile*-associated diarrhea using bacterial therapy. The roles of FMT in other intestinal and metabolic diseases such as ulcerative colitis, Crohn’s disease, and obesity were explored from 2015 to 2017. In recent years, due to the promotion of FMT application, researchers have paid more attention to clinical practice guidelines and treatment strategies. This indicates that the current research is more committed to solving the practical problems in the implementation of FMT.

**Table 5:** Top 30 Keywords related to FMT with the strongest citation bursts.

Keywords	Year	Strength	Begin	End	2012 - 2021
bacteriotherapy	2012	20.05	2012	2017	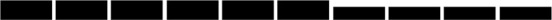
clostridium difficile	2012	18.94	2012	2016	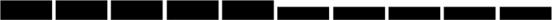
diarrhea	2012	12.55	2012	2015	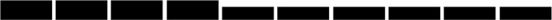
flora	2012	11.3	2012	2015	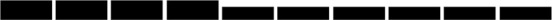
coliti	2012	6.34	2012	2014	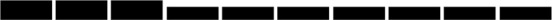
epidemiology	2012	5.57	2012	2016	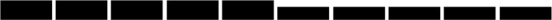
antibiotic associated diarrhea	2012	4.33	2012	2017	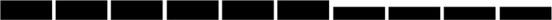
enterocoliti	2012	3.97	2012	2013	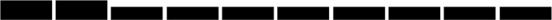
double blind	2012	3.3	2012	2014	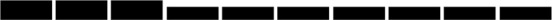
crohns disease	2012	12.91	2013	2017	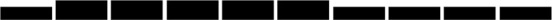
therapy	2012	8.79	2013	2015	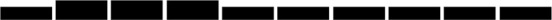
recurrent clostridium difficile	2012	4.68	2013	2018	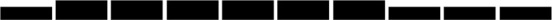
microbiota transplantation	2012	4.28	2013	2016	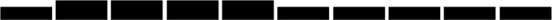
inflammatory bowel disease	2012	4	2013	2015	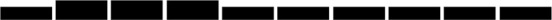
toxin a	2012	3.8	2013	2018	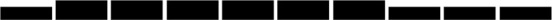
randomized controlled trial	2012	6.9	2014	2018	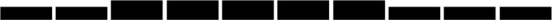
pattern	2012	4.17	2014	2016	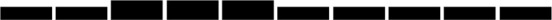
term follow up	2012	4.05	2014	2018	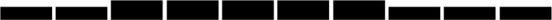
immune system	2012	5.3	2015	2017	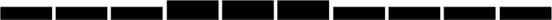
intestinal microbiome	2012	3.77	2015	2016	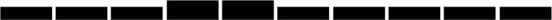
diet induced obesity	2012	3.32	2015	2017	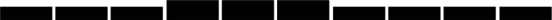
ulcerative coliti	2012	4.19	2016	2017	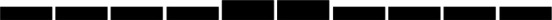
randomized clinical trial	2012	4.18	2016	2017	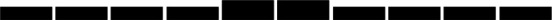
stem cell transplantation	2012	3.79	2016	2018	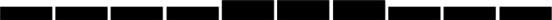
sequence	2012	4.14	2017	2018	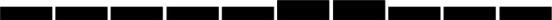
united states	2012	3.78	2017	2018	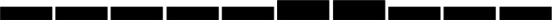
innate immunity	2012	3.78	2017	2018	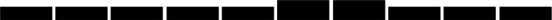
carriage	2012	3.73	2017	2019	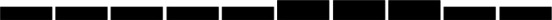
clinical practice guideline	2012	4	2019	2021	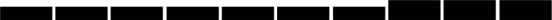
strategy	2012	3.66	2019	2021	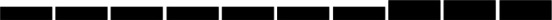

### Analysis of cited authors and co-cited references

**Figure 6:**
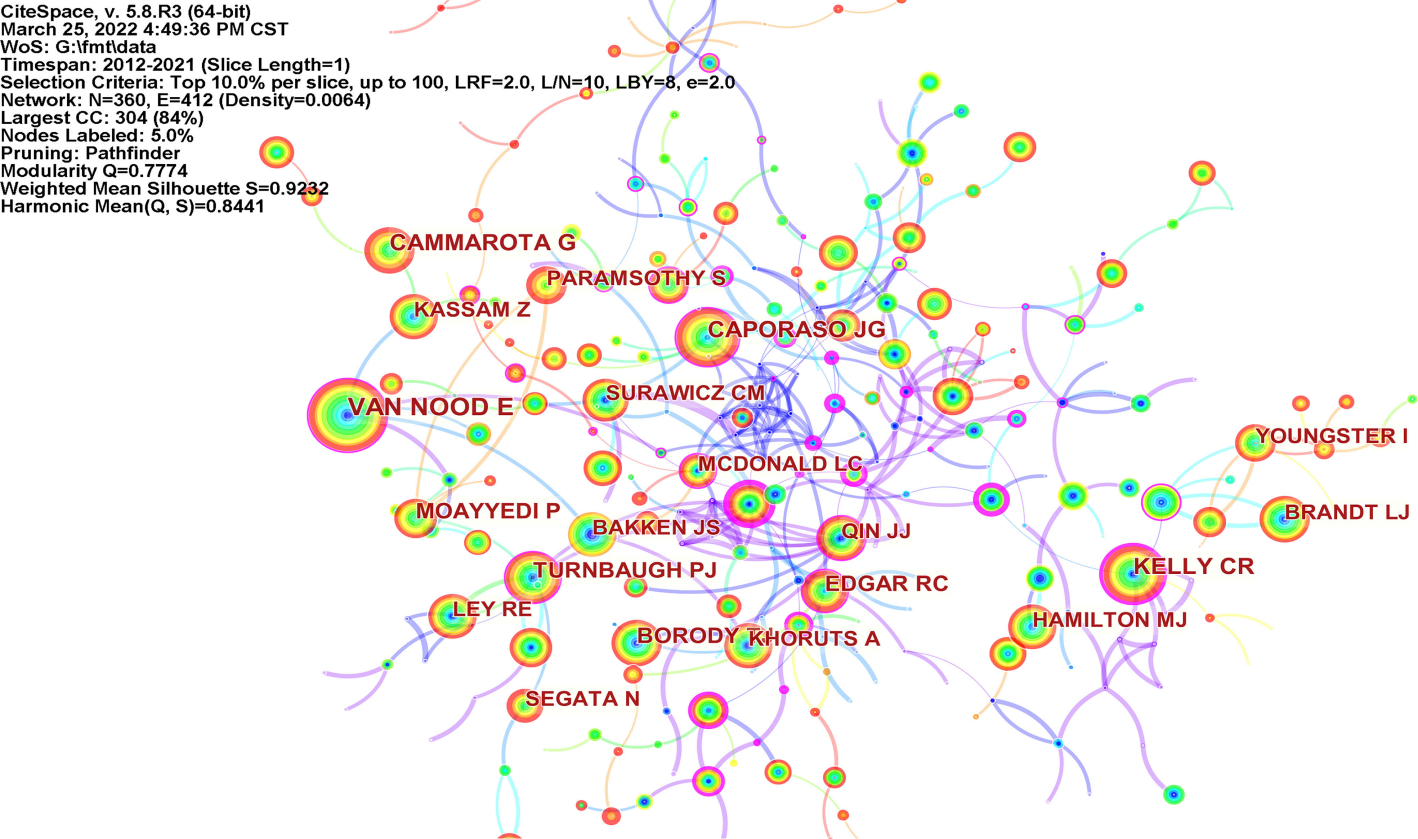
Map of cited authors performing FMT-related research (N=360, E=412).

As shown in **Figure 6** and **Table 6**, the authors with the most citations were Van Nood E, Caporaso JG, and Cammarota G. The most central authors were Sekirov I (0.58), Damman CJ (0.53), and De Leon LM (0.49). Van Nood, from the Academic Medical Center in Amsterdam, the Netherlands, systematically reviewed the treatment of recurrent CDI with donor feces as early as 2009 (Van Nood et al., 2009).

**Table 6:** Top 10 cited authors related to FMT research.

Rank	Count	Centrality	Year	Cited authors
1	543	0.11	2012	Van Nood E
2	363	0.12	2012	Caporaso JG
3	356	0.03	2015	Cammarota G
4	344	0.21	2012	Kelly CR
5	310	0.1	2012	Turnbaugh PJ
6	276	0.08	2015	Moayyedi P
7	262	0.15	2013	Edgar RC
8	245	0	2012	Borody TJ
9	242	0	2012	Hamilton MJ
10	235	0.05	2013	Kassam Z

**Figure 7:**
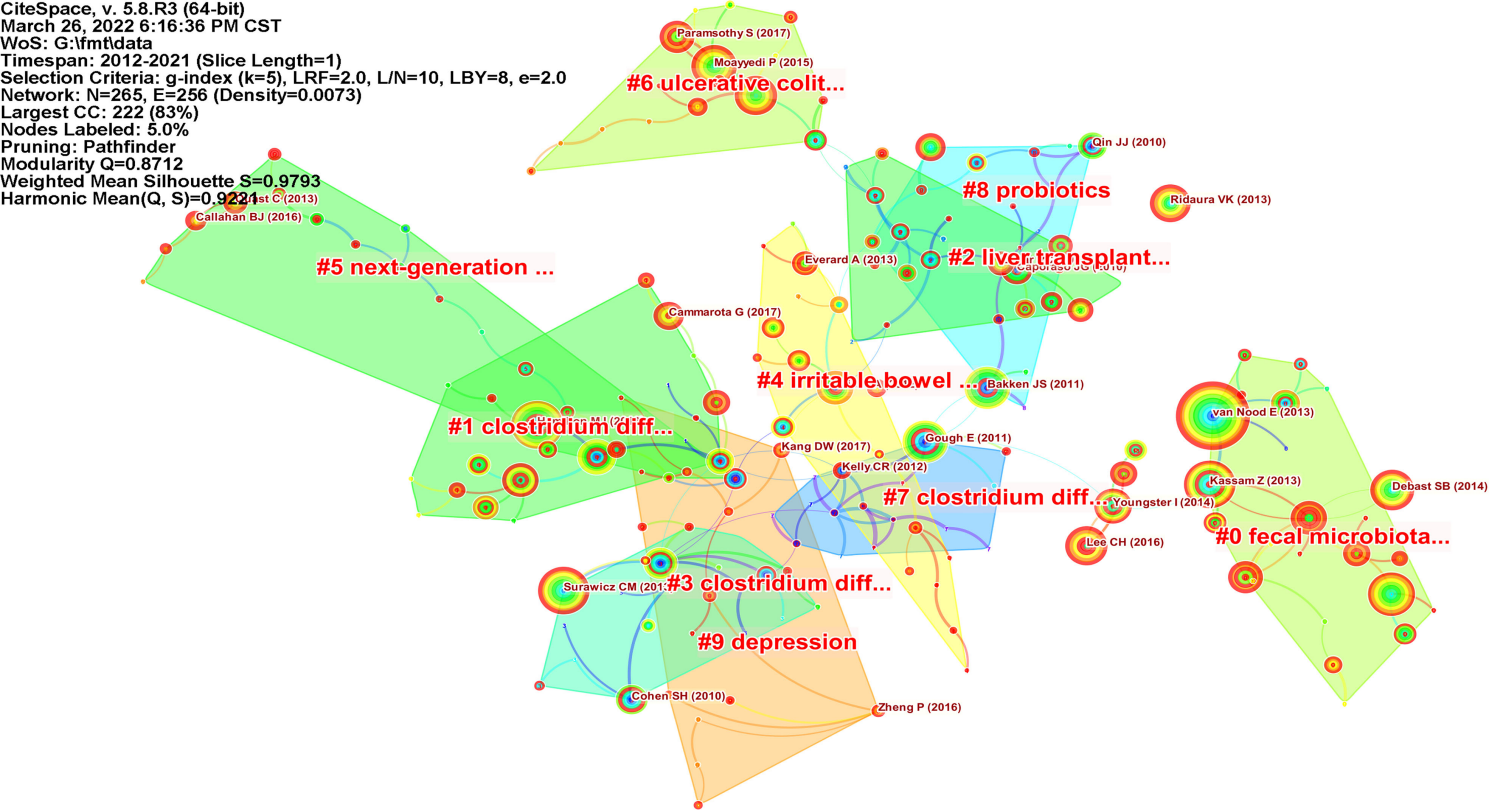
Map of co-cited references related to FMT (N=265, E=256).

The visualization map of co-cited references is shown in **Figure 7**, and the top 10 most frequently cited articles are shown in **Table 7**. These citations are mainly focused on the technology of FMT, the application of FMT in a variety of intestinal diseases (randomized controlled trials), and expert consensus and guidelines. Van Nood and colleagues validated the feasibility of duodenal infusion of donor feces for the treatment of recurrent CDI diarrhea in a randomized controlled trial (Van Nood et al., 2013), which was published in *the New England Journal of Medicine*. This study demonstrated the advantages of FMT over antibiotic intervention. The article with the highest centrality (0.7) was reported by Vrieze and colleagues in 2012. It demonstrated that the transplantation of intestinal microflora from lean human donors could improve insulin sensitivity in recipients (Vrieze et al., 2012), and further cemented the potential role of FMT in obesity treatment. The most frequently cited articles were from *PLoS One*, *Nature*, *Gastroenterology*, *Gut*, and *the New England Journal of Medicine*.

**Table 7:** Top 10 cited references related to FMT research.

Rank	Count	Centrality	Year	Article title	Author	Journal
1	523	0.07	2013	Duodenal infusion of donor feces for recurrent Clostridium difficile	van Nood E	New Engl J Med
2	223	0.04	2015	Fecal Microbiota Transplantation Induces Remission in Patients With Active Ulcerative Colitis in a Randomized Controlled Trial	Moayyedi P	Gastroenterology
3	188	0.01	2013	Guidelines for diagnosis, treatment, and prevention of Clostridium difficile infections	Surawicz CM	Am J Gastroenterol
4	186	0.38	2013	Fecal microbiota transplantation for Clostridium difficile infection: systematic review and meta-analysis	Kassam Z	Am J Gastroenterol
5	165	0	2017	Multidonor intensive faecal microbiota transplantation for active ulcerative colitis: a randomised placebo-controlled trial	Paramsothy S	Lancet
6	164	0.04	2012	Standardized frozen preparation for transplantation of fecal microbiota for recurrent Clostridium difficile infection	Hamilton MJ	Am J Gastroenterol
7	159	0.22	2011	Treating Clostridium difficile infection with fecal microbiota transplantation	Bakken JS	Clin Gastroenterol H
8	159	0.01	2017	European consensus conference on faecal microbiota transplantation in clinical practice	Cammarota G	Gut
9	158	0.03	2015	Burden of Clostridium difficile infection in the United States	Lessa FC	New Engl J Med
10	154	0.59	2011	Systematic review of intestinal microbiota transplantation (fecal bacteriotherapy) for recurrent Clostridium difficile infection	Gough E	Clin Infect Dis

Clustering analysis was performed on the co-cited references (**Figure 7**), and the top 10 clusters were identified (**Table 8**). The development of sequencing technologies and the deepening of research on the composition and function of intestinal microorganisms, has led to an expansion in the scope of FMT research. Liver transplantation (cluster #2) and irritable bowel syndrome (cluster #4) had 19 and 18 co-cited references, respectively. Additionally, the therapeutic effect of FMT on psychological diseases such as depression has attracted extensive attention.

**Table 8:** Top 10 clusters of cited references related to FMT research.

ClusterID	Size	Sihouette	Year	Label (LLR)
#0	23	1	2015	fecal microbiota transplant
#1	20	0.986	2014	clostridium difficile infection
#2	19	1	2011	liver transplantation
#3	19	0.958	2011	clostridium difficile
#4	18	0.931	2016	irritable bowel syndrome
#5	18	1	2012	next-generation sequencing
#6	16	1	2016	ulcerative colitis
#7	16	0.99	2010	clostridium difficile
#8	14	0.944	2009	probiotics
#9	14	1	2016	depression

## Discussion

This study represents the first bibliometric and visual analysis of FMT research over the past 10 years. We used CiteSpace to perform a bibliometric analysis on FMT studies that were carried out between 2012 and 2021, and the current status and future trends of FMT research were identified through the analysis of the distribution of countries, institutions, journals and authors, as well as core keywords and references. As research interest into the effects of intestinal microorganisms has increased, FMT has attracted continuous and increasing attention as a potential therapeutic intervention. Our data shows that publications and citations related to FMT increase yearly, and it has become a hot research topic. The USA contributed the most significant number of publications, becoming one of the main driving forces in FMT research. American institutions have extensive resources in universities, medical institutions, and social organizations. European and American countries have the highest degree of centralization. They occupy a dominant position, demonstrating that they have advantages in international exchanges and cooperation. FMT research is also showing strong momentum in China. In recent years, Nanjing Medical University has committed to the standardization of FMT (Cui et al., 2015; Zhang et al., 2018). The Medical College of Zhejiang University has focused on the use of animal models of FMT to verify the role of specific bacteria in disease processes (Li et al., 2017; Bian et al., 2019). These institutions have published a large amount of literature, which will provide valuable scientific evidence and practical guidance for FMT moving forward.

Alexander Khoruts was identified as the most productive author. He was also the lead author of the clinical therapeutic guidelines for CDI, a study that was cited more than 500 times (Bakken et al., 2011). His research revealed that the mechanism of FMT is related to the restoration of normal intestinal microbial community structure and function (Khoruts and Sadowsky, 2016; Khoruts et al., 2021). In addition, he also investigated the use of FMT in treating liver cirrhosis, autism, and other diseases (Kang et al., 2017; Cheng et al., 2021). Faming Zhang published 35 articles on FMT studies. The development of the intelligent fecal bacteria isolation system, and the technology of washing microbiota transplantation, were led by his team. These technologies improve the safety of FMT and reduce the probability of adverse events (Zhang et al., 2020). This study was published in *Protein & Cell* and featured on the cover, pushing FMT into a new development stage. Additionally, the widely cited study carried out by Van Noude and colleagues demonstrating significant advantages of infusion of donor feces for treatment of recurrent CDI over traditional vancomycin therapy (Van Nood et al., 2013), also pushed the field forward. These authors have a strong presence in FMT research and have contributed to the exploration of safety, efficacy, and operability.

*C. difficile* is a gram-positive anaerobic bacteria (Chen et al., 2021), which can cause gastrointestinal infections (Zhou et al., 2021). *C. difficile* was first discovered in 1935 as normal flora in the intestines of newborn babies (Hopkins and Wilson, 2018). Use of antibiotics can cause *C. difficile* proliferation, which in turn leads to refractory diseases such as severe diarrhea, pseudomembranous colitis, and intestinal obstruction. However, antibiotic treatment seems to be ineffective against CDI, and may even cause antibiotic resistance (Spigaglia et al., 2018). An article in *Lancet* reported the first case of FMT for CDI treatment in 1983 (Schwan et al., 1983), which pioneered a new approach to non-antibiotic therapy. To date, FMT remains the recommended treatment for patients with severe and fulminant CDI according to ACG clinical guidelines (Kelly et al., 2021). From 2012 to 2021, the number of articles with the keywords of *Clostridium difficile* infection and *Clostridium difficile* was 306 and 189, respectively. We believe that the treatment of CDI remains the main direction of clinical and basic research on FMT.

Keywords are considered to reflect high-frequency hot spots in a particular field (Lin et al., 2022). We also conducted a comprehensive analysis of the co-cited references and categories, and summarized the research hot spots as follows: 1) Inflammatory bowel disease and CDI (Cammarota et al., 2017), principally including ulcerative colitis (Costello et al., 2019; Matsuoka, 2021), and Crohn’s disease (Bak et al., 2017; Sokol et al., 2020), also including constipation (Ge et al., 2017; Zhang et al., 2021), and diarrhea (Dai et al., 2019); 2) Complementary and alternative treatment of several metabolic diseases, such as obesity (Allegretti et al., 2020; Yu et al., 2020), type 2 diabetes (Wang et al., 2019a), metabolic syndrome (Mocanu et al., 2021); 3) Adjuvant tumor immunotherapy, mainly to reduce drug toxicity and modulate the immune response (Chang et al., 2020; Baruch et al., 2021); 4) Developmental disorders and psychological diseases, mainly related to the treatment of children’s autism (Kang et al., 2019) and the relief of symptoms of depression and anxiety (Kilinçarslan and Evrensel, 2020; Rao et al., 2021); 5) Transplantation mode and action mechanisms, such as *in vitro* sorting of transplanted microorganisms (Zhang et al., 2020), and short-chain fatty acid and bile acid metabolism (Seekatz et al., 2018); 6) Potential indications and risk assessment (Lagier and Raoult, 2016; Gupta et al., 2021), and the utilization of standardized fecal sample banks (Kragsnaes et al., 2020).

Emerging trends can be identified through the analysis of burst keywords (Chen et al., 2014). Burst keywords at a specific time often mean that research in related fields has attracted attention. Based on the burst keywords in recent years, we believe that the establishment and improvement of clinical guidelines for FMT will become an academic trend. This will help clarify the indications, reduce potential risks, and develop optimum treatment strategies. Although extensive clinical studies have proved the safety of FMT (Goloshchapov et al., 2019; Saha et al., 2021), a small number of patients undergoing FMT may suffer from adverse reactions such as abdominal pain, diarrhea (Michailidis et al., 2021), recurrent infection, and even death (Ser et al., 2021). Clinical guidelines are the evidence base for medical practice (Kondylakis et al., 2020), and therefore, are important for guiding research and promotion of FMT (Haifer et al., 2020), operating procedures (Cammarota et al., 2019), and administration routes (Ross and Reveles, 2020; Halaweish et al., 2022). At the policy and legislative levels, there is a need for both a flexible regulatory framework to promote the application and research, and traceability of fecal donors to ensure patient safety (Vyas et al., 2015; Cammarota et al., 2019). Additionally, ethical issues raised by fecal donation also need to be addressed (Grigoryan et al., 2020; Hollingshead et al., 2021).

In this study, we used CiteSpace to reveal the cutting-edge hot spots and dynamic changes in the FMT research field over the past decade. Our study found that FMT can improve the intestinal microenvironment and has prominent advantages in the treatment of many diseases. However, the large-scale application of FMT also faces safety, legal, and regulatory challenges. Furthermore, there are some limitations to this study. Firstly, all articles were retrieved from the Web of Science Core Collection only. Secondly, search strategies are not guaranteed to cover all FMT-related articles. Thirdly, due to the dynamic database updates and the rapid development of FMT research, our study cannot include all of the latest findings.

## Conclusion

From 2012 to 2021, publications and citations relating to FMT research increased year by year. The USA has played a leading role in the field, with Harvard Medical School and Alexander Khoruts being the most prolific institutions and authors, respectively. A few key studies have pushed FMT into the spotlight, including the widely cited and influential study from Noude and colleagues titled “Duodenal infusion of donor feces for recurrent *Clostridium difficile*”. In recent years, the research of Chinese scholars has advanced at a rapid pace. Research hotspots and trends mainly involve the treatment of digestive system and other system diseases, with a particular focus on CDI and ulcerative colitis. In addition, new indications, new technologies, and new norms of FMT are also focuses of research. Overall, this study reports the advances in the field over the past 10 years from multiple dimensions through visual analysis of FMT research, paving the way for the follow-up research.

## Data availability statement

The original contributions presented in the study are included in the article/**Supplementary Material**. Further inquiries can be directed to the corresponding author.

## Author contributions

Conceptualization: LZ and MY; methodology: FZ and PY; software: YC; formal analysis: JW; data curation: LZ; writing—original draft preparation: FZ and PY; writing—review and editing: RW and BL; visualization: FZ, PY and YC; supervision: MY; project administration: LZ; funding acquisition: LZ. All authors have read and agreed to the published version of the manuscript.

## Funding

This work is supported by the National Natural Science Foundation of China (grant number: 81973730).

## Conflict of interest

The authors declare that the research was conducted in the absence of any commercial or financial relationships that could be construed as a potential conflict of interest.

## Publisher’s note

All claims expressed in this article are solely those of the authors and do not necessarily represent those of their affiliated organizations, or those of the publisher, the editors and the reviewers. Any product that may be evaluated in this article, or claim that may be made by its manufacturer, is not guaranteed or endorsed by the publisher.

## Supplementary material

Supplementary Figure 1 Flowchart of retrieval process.

## Abbreviations

FMT, fecal microbiota transplantation; CDI, *Clostridium difficile* infection; *C. difficile*, *Clostridium difficile;* IF, impact factor.
