# Supplementary figures and images for: Bibliometric and visual analysis of fecal microbiota transplantation research from 2012 to 2021
Source: Front Cell Infect Microbiol. 2022 Nov 10;12:1057492. doi: 10.3389/fcimb.2022.1057492 (PMC9684174; doi:10.3389/fcimb.2022.1057492)

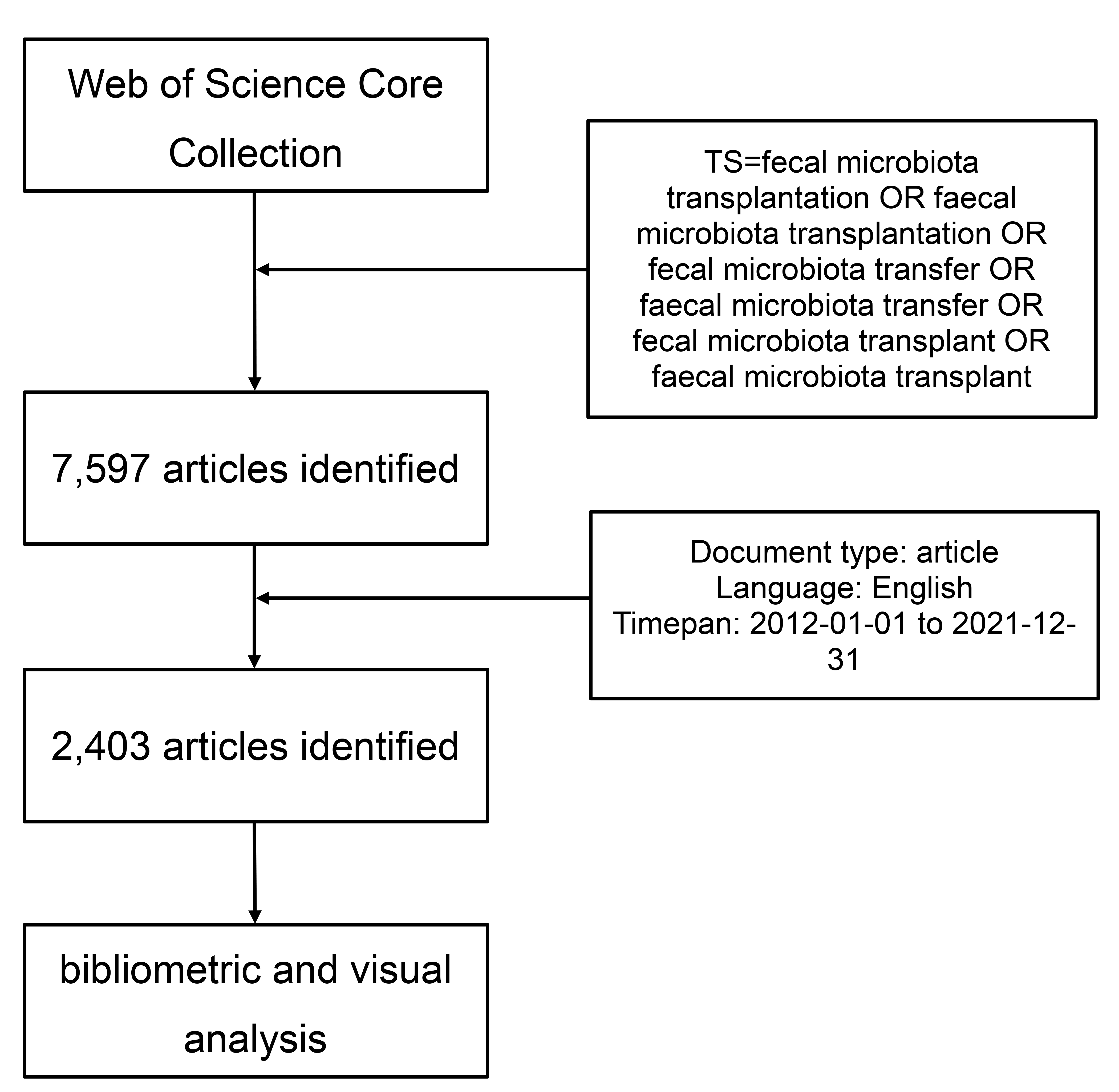

Supplement: Supplementary Figure 1 — Flowchart of retrieval process. [file Image_1.tif]
